# Survival analysis of cancer patients using a new extended Weibull distribution

**DOI:** 10.1371/journal.pone.0264229

**Published:** 2022-02-23

**Authors:** Hadeel S. Klakattawi

**Affiliations:** Department of Statistics, Faculty of Science, King Abdulaziz University, Jeddah, Saudi Arabia; University of Bradford, UNITED KINGDOM

## Abstract

One of the most important applications of statistical analysis is in health research and applications. Cancer studies are mostly required special statistical considerations in order to find the appropriate model for fitting the survival data. Existing classical distributions rarely fit such data well and an increasing interest has been shown recently in developing more flexible distributions by introducing some additional parameters to the basic model. In this paper, a new five-parameters distribution referred as alpha power Kumaraswamy Weibull distribution is introduced and studied. Particularly, this distribution extends the Weibull distribution based on a novel technique that combines two well known generalisation methods, namely, alpha power and T-X transformations. Different characteristics of the proposed distribution, including moments, quantiles, Rényi entropy and order statistics are obtained. The method of maximum likelihood is applied in order to estimate the model parameters based on complete and censored data. The performance of these estimators are examined via conducting some simulation studies. The potential importance and applicability of the proposed distribution is illustrated empirically by means of six datasets that describe the survival of some cancer patients. The results of the analysis indicated to the promising performance of the alpha power Kumaraswamy Weibull distribution in practice comparing to some other competing distributions.

## 1 Introduction

Many statistical distributions have been extensively utilized for analysing time to event data also referred to as survival or reliability data, in different areas of applicability, including the medical field. Medical scientists are mostly interested in studying the survival of patients with cancer in the applied research. These research are most often require special statistical attentions and adjustments in the context of finding and choosing the appropriate model that accurately determine and estimate the survival data and yielded in reliable results and valid inferences. It is possible to consider the Weibull distribution [1], to be one of the most popular distributions for modeling such data that explain the mortality and failure. However, the classical two-parameter Weibull distribution is less suitable for fitting when data show non-monotonic failure rates due to its limitation in modeling only monotonically increasing and decreasing hazard functions. Therefore, there is a crucial need in many cases to enhance the traditional Weibull for modeling biomedical data. It follows that many attempts have been made to extend the baseline Weibull model by adding one or more additional parameters to achieve more flexibility in generating different shapes of data. To illustrate, [2] suggested the exponentiated Weibull distribution by applying the exponentiated method [3] in which a shape parameter is added to a baseline distribution. Beta-Weibull ditribution [4] is introduced based on the beta-generated method by [5]. Marshall–Olkin extended Weibull distribution [6] has been suggested to modify the Weibull distribution using the technique by [7]. This distribution has been applied to fit a dataset representing the remission times of bladder cancer patients. Furthermore, the Maxwell–Weibull distribution is introduced by [8] to model lifetime data. On the basis of the zero truncated Poisson model, [9] proposed a new compound distribution called the quasi Poisson Burr X exponentiated Weibull distribution, which accommodated many important failure rates. Moreover, in a recent study, [10] have derived a bimodal form of the Weibull distribution.

Researchers have shown a keen interest in developing new methods for expanding lifetime distributions. [11] developed a new method that add two extra shape parameters *a*, *b* > 0 to an arbitrary baseline distribution, called Kumaraswamy generalized (Kum-G) with a cumulative distribution function (cdf) defined as

$$\begin{array}{c} F^{Kum-G}\left(x;a,b,\theta \right)=1-{\left[1-{\left(G\left(x;\theta \right)\right)}^{a}\right]}^{b}, \end{array}$$

where *X* is a continuous random variable whose baseline (cdf) is *G*(*x*;***θ***) with a vector of parameter(s) ***θ***. A number of studies have been applied this method to develop new distribution such as, the Kumaraswamy Gumbel by [12], the Kumaraswamy Birnbaum-Saunders by [13], the Kumaraswamy Burr XII distribution by [14], the Kumaraswamy generalized Rayleigh distribution by [15], the Kumaraswamy Laplace distribution by [16], the Kumaraswamy half-logistic distribution by [17], the Kumaraswamy exponentiated Weibull by [18], the Kumaraswamy Marshall-Olkin exponential distribution by [19] and the Kumaraswamy Pareto IV distribution by [20], among others.

[21] introduced the Kumaraswamy Weibull (KumW) distribution as a generalization of the Weibull distribution and demonstrated its flexibility to fit failure data. The proposed distribution can be obtained by assuming $G\left(x\right)=1-e^{-{\left(\lambda x\right)}^{c}}$ of the Weibull distribution with scale parameter λ > 0 and shape parameter *c* > 0. Thus, the cdf and probability density function (pdf) of the KumW is obtained respectively as

$$\begin{array}{c} F^{KumW}\left(x;a,b,c,\lambda \right)=1-{\left[1-{\left(1-e^{-{\left(\lambda x\right)}^{c}}\right)}^{a}\right]}^{b}, \end{array}$$ (1)

and

$$\begin{array}{c} f^{KumW}\left(x;a,b,c,\lambda \right)=abc\lambda^{c}x^{c-1}e^{-{\left(\lambda x\right)}^{c}}{\left(1-e^{-{\left(\lambda x\right)}^{c}}\right)}^{a-1}{\left[1-{\left(1-e^{-{\left(\lambda x\right)}^{c}}\right)}^{a}\right]}^{b-1}. \end{array}$$ (2)

The KumW distribution has been considered by some authors, for example, [22, 23] discussed different types of statistical inference for constant stress accelerated life tests based on censored sampling data from the KumW distribution. [24] discussed some Bayesian analyses for the KumW distribution. [25] considered a regression model for bivariate random variables based on the bivariate KumW distribution. Although the KumW has been perfectly described many datasets, it has been modified by some authors. For instance, [26] in which the KumW is generalised by considering the new modified Kumaraswamy-G in [27]. Additionally, [28] who consider the exponentiated class in [3] to generalise the KumW. More recently, [29] generalised the KumW by considering the transmuted class in [30].

On the other hand, [31] suggested a new approach, called alpha power transformation (APT), for generating distributions with additional parameter *α* in order to add more flexibility. Then, the APT for an arbitrary baseline cdf *G* and pdf *g* for a random variable *X* with a vector of parameter(s) ***θ*** can be obtained as follows

$$\begin{array}{c} F^{APT}\left(x;\alpha ,\theta \right)=\{\begin{array}{cc} \frac{\alpha^{G(x;\theta )}-1}{\alpha -1} & \text{if}\;\alpha >0,\alpha \neq 1\\ G(x;\theta ) & \text{if}\;\alpha =1, \end{array} \end{array}$$ (3)

with the corresponding pdf as

$$\begin{array}{c} f^{APT}\left(x;\alpha ,\theta \right)=\{\begin{array}{cc} \frac{log\alpha }{\alpha -1}g\left(x;\theta \right)\alpha^{G(x;\theta )} & \text{if}\;\alpha >0,\alpha \neq 1\\ g(x;\theta ) & \text{if}\;\alpha =1. \end{array} \end{array}$$ (4)

[31] applied their suggested way to a one-parameter exponential distribution to develop alpha power exponential distribution with two-parameters. Several authors have been applied the method of APT to extend some exiting distributions in the literature. Examples include the alpha power Weibull distribution by [32, 33], the alpha-power inverse Weibull distribution by [34, 35], the alpha power inverted exponential by [36], the alpha power transformed extended exponential distribution by [37], the alpha power transformed power Lindley by [38], the alpha power transformed Lindley by, [39], the alpha power transformed inverse Lindley by [40], the alpha power transformed inverse Lomax by [41], alpha power Maxwell distribution by [42], the alpha power exponentiated inverse Rayleigh by [43] and the alpha power Weibull–exponential by [44], among others.

Motivated by the idea that developing some new distributions will eliminate some issues that inherent in the existing distributions, the main objective of this paper is to introduce a novel generalization for the Weibull distribution. This distribution is constructed by combining the works of [21, 31] introducing a new five-parameter distribution refereed to as the alpha power Kumaraswamy Weibull (APKumW) distribution. As compared to other probability distributions presented in the literature, the proposed model will increase the flexibility and adaptability for describing different shapes of hazard-rate functions, such as decreasing, increasing, bath-tub and upside down bath-tub shaped, which might extensively experienced in real life data. Particularly, as indicated by [45] for the effectiveness of employing the APT distributions for cancer research, this paper focuses in exploring the adaptability of the proposed distribution to describe the survival time by analyzing some cancer datasets. Additionally, another objective is to estimate the unknown model parameters using maximum likelihood method for both complete and censored cancer datasets.

The rest of the paper is organized as follows. In Section 2, the APKumW distribution is defined and its special cases are presented along with an useful expansion for its pdf. In Section 3, some of the properties of the proposed distribution are discussed. The maximum likelihood estimators (MLEs) of the distribution parameters are obtained in Section 4 based on uncensored and censored data. Consequently, some different simulation studies are carried out to assess the performance of the MLEs in Section 5. Finally, different applications of the APKumW distribution to complete and censored datasets are presented in Section 6. All computations throughout this paper were performed using the statistical programming language R.

## 2 Alpha power Kumaraswamy Weibull distribution

The APKumW distribution is suggested in this paper based on substituting by Eqs (1) and (2) respectively in Eqs (3) and (4). That is, the random variable *X* is said to have the APKumW distribution with five parameters ***θ*** = {*a*, *b*, *c*, λ, *α*}, if the cdf of *X* is

$$\begin{array}{c} F\left(x;\theta \right)=\{\begin{array}{cc} \frac{\alpha^{1-{\left[1-{\left(1-e^{-{\left(\lambda x\right)}^{c}}\right)}^{a}\right]}^{b}}-1}{\alpha -1} & \text{if}\;\alpha >0,\alpha \neq 1\\ 1-{\left[1-{\left(1-e^{-{\left(\lambda x\right)}^{c}}\right)}^{a}\right]}^{b} & \text{if}\;\alpha =1, \end{array} \end{array}$$ (5)

and its corresponding pdf is

$$\begin{array}{c} f\left(x;\theta \right)=\{\begin{array}{cc} \begin{array}{cc} \frac{log(\alpha )}{\alpha -1} & abc\lambda^{c}x^{c-1}e^{-{\left(\lambda x\right)}^{c}}{\left(1-e^{-{\left(\lambda x\right)}^{c}}\right)}^{a-1}\times \\ & {\left[1-{\left(1-e^{-{\left(\lambda x\right)}^{c}}\right)}^{a}\right]}^{b-1}\alpha^{1-{\left[1-{\left(1-e^{-{\left(\lambda x\right)}^{c}}\right)}^{a}\right]}^{b}} \end{array} & \text{if}\;\alpha >0,\alpha \neq 1\\ abc\lambda^{c}x^{c-1}e^{-{\left(\lambda x\right)}^{c}}{\left(1-e^{-{\left(\lambda x\right)}^{c}}\right)}^{a-1}{\left[1-{\left(1-e^{-{\left(\lambda x\right)}^{c}}\right)}^{a}\right]}^{b-1} & \text{if}\;\alpha =1. \end{array} \end{array}$$ (6)

Additionally, the survival and hazard rate functions of the APKumW distribution are respectively given by

$$\begin{array}{c} SF\left(x;\theta \right)=\{\begin{array}{cc} \frac{\alpha }{\alpha -1}(1-\alpha^{-{\left[1-{\left(1-e^{-{\left(\lambda x\right)}^{c}}\right)}^{a}\right]}^{b}}) & \text{if}\;\alpha >0,\alpha \neq 1\\ {\left[1-{\left(1-e^{-{\left(\lambda x\right)}^{c}}\right)}^{a}\right]}^{b} & \text{if}\;\alpha =1, \end{array} \end{array}$$ (7)

and

$$\begin{array}{c} h\left(x;\theta \right)=\{\begin{array}{cc} \begin{array}{c} log(\alpha )abc\lambda^{c}x^{c-1}e^{-{\left(\lambda x\right)}^{c}}{\left(1-e^{-{\left(\lambda x\right)}^{c}}\right)}^{a-1}\times \\ {\left[1-{\left(1-e^{-{\left(\lambda x\right)}^{c}}\right)}^{a}\right]}^{b-1}\frac{\alpha^{-{\left[1-{\left(1-e^{-{\left(\lambda x\right)}^{c}}\right)}^{a}\right]}^{b}}}{1-\alpha^{-{\left[1-{\left(1-e^{-{\left(\lambda x\right)}^{c}}\right)}^{a}\right]}^{b}}} \end{array} & \text{if}\;\alpha >0,\alpha \neq 1\\ abc\lambda^{c}x^{c-1}e^{-{\left(\lambda x\right)}^{c}}\frac{{\left(1-e^{-{\left(\lambda x\right)}^{c}}\right)}^{a-1}}{1-{\left(1-e^{-{\left(\lambda x\right)}^{c}}\right)}^{a}} & \text{if}\;\alpha =1. \end{array} \end{array}$$ (8)

**Fig 1:**
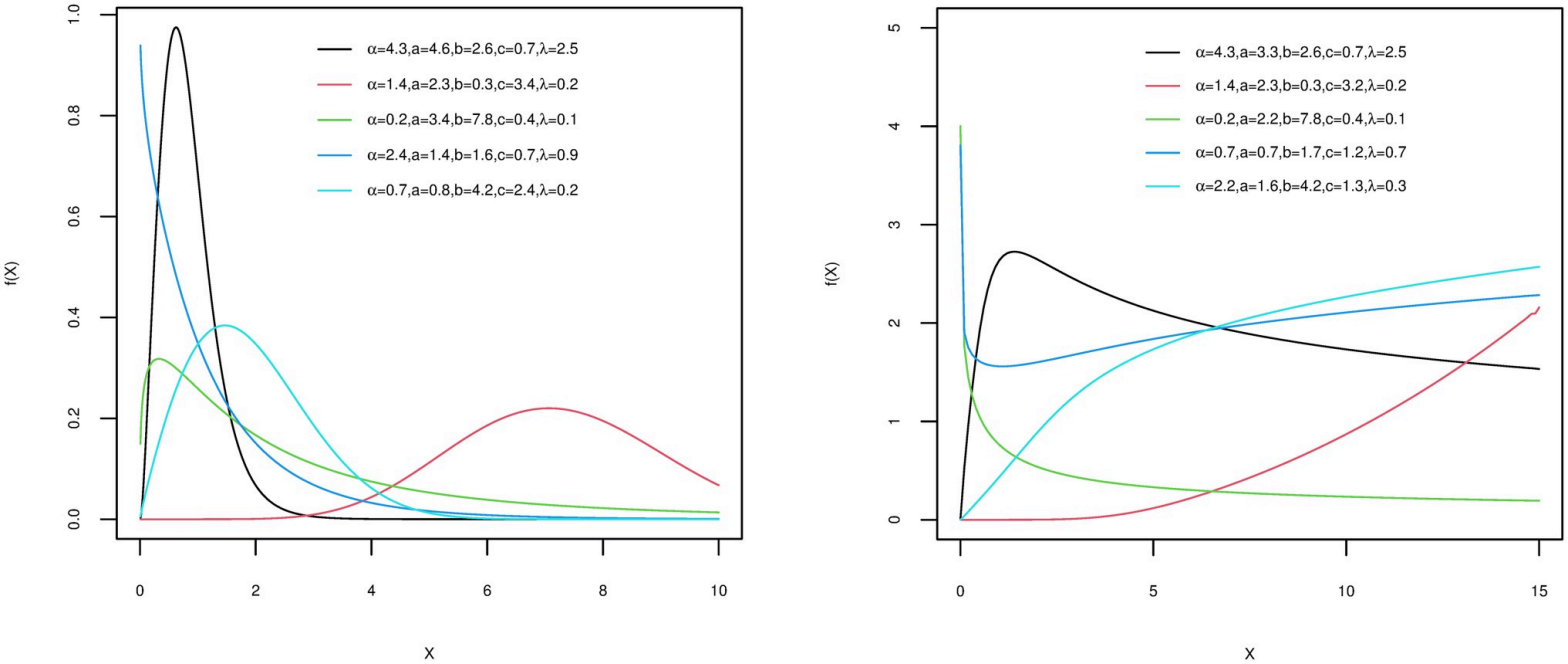
The APKumW pdf and hazard function for various values of its parameters.

Incorporating skewness to the base distribution is done by adding the parameter *α*. The APKumW model is therefore a suitable model to describe positively skewed patterns in biomedical and public health data. Fig 1 displays some of the shapes that the pdf and hazard functions of the APKumW distribution can take for different values of its parameters. These different behaviours indicate the flexibility and adaptability for the APKumW to fit a variety of data shapes.

### 2.1 Special cases

Table 1 shows important special models of the APKumW distribution.

**Table 1:** Special models of the APKumW distribution.

*α*	a	b	c	λ	Resulting Distribution
1	−	−	−	−	KumW
−	−	−	1	−	APKumExp
−	−	−	2	−	APKum-Rayleigh
−	1	1	1	−	AP-Exponential
−	1	1	2	−	AP-Rayleigh
−	1	1	−	−	AP-Weibull
1	−	1	−	−	Expontiated Weibull
1	−	1	2	−	Expontiated Rayleigh
1	−	1	1	−	Expontiated Exponential
1	1	1	1	−	Exponential
1	1	1	1	−	Rayleigh
1	1	1	−	−	Weibull

### 2.2 Expansion of the probability density function

Using the following power series expansion

$$\begin{array}{c} \alpha^{-z}=\sum_{k=0}^{\infty }\frac{{\left(-log\left(\alpha \right)\right)}^{k}z^{k}}{k!}, \end{array}$$ (9)

the pdf in Eq (6) can be written as

$$\begin{array}{c} \begin{array}{cc} f\left(x;\theta \right)=\frac{\alpha }{\alpha -1} & abc\lambda^{c}x^{c-1}e^{-{\left(\lambda x\right)}^{c}}{\left(1-e^{-{\left(\lambda x\right)}^{c}}\right)}^{a-1}\times \\ \sum_{k=0}^{\infty } & \frac{{\left(-1\right)}^{k}}{k!}{\left(log(\alpha )\right)}^{k+1}{\left[1-{\left(1-e^{-{\left(\lambda x\right)}^{c}}\right)}^{a}\right]}^{bk+b-1}. \end{array} \end{array}$$

Then, the following binomial expansion

(1-z)b-1=∑j=0∞(-1)j(b-1j)zj;for|z|<1andb>0, (10)

is applied twice to obtain a useful expansion of the pdf of the APKumW as follows

f(x;θ)=αα-1abcλc∑k=0∞∑j=0∞∑i=0∞(-1)i+j+kk!(b(k+1)-1j)(a(j+1)-1i)(log(α))k+1xc-1e-(i+1)(λx)c. (11)

## 3 Properties of alpha power Kumaraswamy Weibull distribution

Some properties of the APKumW distribution are considered in the following as

### 3.1 Simulation, quantiles and median

To simulate a random variable from APKumW distribution, Eq (5) can be used to obtain

$$\begin{array}{c} X=\frac{1}{\lambda }{\left[-log\{1-{\left[1-{\left(1-\frac{log(U(\alpha -1)+1)}{log(\alpha )}\right)}^{\frac{1}{b}}\right]}^{\frac{1}{a}}\}\right]}^{\frac{1}{c}}, \end{array}$$ (12)

where *U* is a random variable follows a uniform (0, 1) distribution. Also, the *p*^*th*^ quantile function of the APKumW distribution for 0 < *p* < 1, is given by

$$\begin{array}{c} X_{p}=\frac{1}{\lambda }{\left[-log\{1-{\left[1-{\left(1-\frac{log(p(\alpha -1)+1)}{log(\alpha )}\right)}^{\frac{1}{b}}\right]}^{\frac{1}{a}}\}\right]}^{\frac{1}{c}}. \end{array}$$ (13)

Consequently for $p=\frac{1}{2}$, the median for the APKumW can be obtained as

$$\begin{array}{c} X_{0.5}=\frac{1}{\lambda }{\left[-log\{1-{\left[1-{\left(1-\frac{log(\alpha +1)-log(2)}{log(\alpha )}\right)}^{\frac{1}{b}}\right]}^{\frac{1}{a}}\}\right]}^{\frac{1}{c}}. \end{array}$$ (14)

### 3.2 Moments

The *r*^*th*^ moment of a random variable *X* is given by

$$\begin{array}{c} E\left(X^{r}\right)=\int_{0}^{\infty }x^{r}f\left(x;\theta \right)dx. \end{array}$$

Then, the *r*^*th*^ moment of the APKumW is given from Eq (11) as

E(Xr)=αα-1abcλc∑k=0∞∑j=0∞∑i=0∞(-1)i+j+kk!(b(k+1)-1j)(a(j+1)-1i)(log(α))k+1×∫0∞xr+c-1e-(i+1)(λx)cdx.

By letting *u* = (*i*+ 1)(λ*x*)^*c*^, then the *r*^*th*^ moment can be obtained as

E(Xr)=αα-1abλr∑k=0∞∑j=0∞∑i=0∞(-1)i+j+kk!(b(k+1)-1j)(a(j+1)-1i)(log(α))k+1×(1i+1)rc+1Γ(rc+1), (15)

where Γ(.) is the gamma function. Subsequently, the mean and variance can be obtained by substituting *r* = 1 and *r* = 2 in Eq (15).

The moment generating function of a random variable *X* can be defined with the form

$$\begin{array}{c} M_{x}\left(t\right)=E\left(e^{tx}\right)=\int_{0}^{\infty }e^{tx}f\left(x;\theta \right)dx. \end{array}$$

That is, using the following power series expansion for the exponential function

$$\begin{array}{c} e^{z}=\sum_{l=0}^{\infty }\frac{{\left(z\right)}^{l}}{l!}, \end{array}$$ (16)

the moment generating function of a random variable *X* whose pdf in Eq (6), can be obtained similarly as

Mx(t)=abαα-1∑l=0∞∑k=0∞∑j=0∞∑i=0∞(-1)i+j+kk!l!(b(k+1)-1j)(a(j+1)-1i)(log(α))k+1×(tλ)l(1i+1)lc+1Γ(lc+1). (17)

### 3.3 Rényi entropy

The Rényi entropy of a random variable *X* represents a measure of variation of the uncertainty and given by

$$\begin{array}{c} RE_{x}\left(\nu \right)=\frac{1}{1-\nu }log(\int_{-\infty }^{\infty }{\left[f(x;\theta )\right]}^{\nu }dx)\;;\;\nu >0,\;\nu \neq 0. \end{array}$$

Then from Eq (6), we have

$$\begin{array}{cc} {\left[f(x;\theta )\right]}^{\nu }= & {\left(\frac{\alpha log\left(\alpha \right)}{\alpha -1}\right)}^{\nu }{\left(abc\lambda^{c}\right)}^{\nu }x^{\nu \left(c-1\right)}e^{-\nu {\left(\lambda x\right)}^{c}}{\left(1-e^{-{\left(\lambda x\right)}^{c}}\right)}^{\nu \left(a-1\right)}\times \\ & \;{\left[1-{\left(1-e^{-{\left(\lambda x\right)}^{c}}\right)}^{a}\right]}^{\nu \left(b-1\right)}\alpha^{-\nu {\left[1-{\left(1-e^{-{\left(\lambda x\right)}^{c}}\right)}^{a}\right]}^{b}}. \end{array}$$

Applying Eqs (9) and (10) twice, we obtain

[f(x;θ)]ν=(αα-1abcλc)νxν(c-1)∑k=0∞∑j=0∞∑i=0∞(-1)i+j+kk!(b(k+ν)-νj)(a(j+ν)-νi)×νk(log(α))ν+ke-(i+ν)(λx)c.

Then,

REx(ν)=11-νlog{(αα-1abcλc)ν∑k=0∞∑j=0∞∑i=0∞(-1)i+j+kk!(b(k+ν)-νj)(a(j+ν)-νi)×νk(log(α))ν+k∫0∞xν(c-1)e-(i+ν)(λx)cdx}.

By assuming *u* = (*i*+ *ν*)(λ*x*)^*c*^, the Rényi entropy for the APKumW can be expressed as

REx(ν)=ν1-νlog(abαα-1)-log(λc)+11-νlog{∑k=0∞∑j=0∞∑i=0∞(-1)i+j+kk!(b(k+ν)-νj)(a(j+ν)-νi)×νk(log(α))ν+k(1i+ν)ν(c-1)c+1cΓ(ν(c-1)c+1c)}. (18)

### 3.4 Order statistics

Suppose that *F*(*x*) and *f*(*x*) are respectively the cdf and pdf of *n* independent and identically distributed random variables *X*_1_, *X*_2_, …*X*_*n*_ with *X*_1:*n*_ < *X*_2:*n*_ < … < *X*_*n*:*n*_ be their corresponding ordered statistics. Then, the pdf of the *s*^*th*^ order statistic can be obtained as

$$\begin{array}{c} f_{s:n}\left(x\right)=\frac{n!}{\left(s-1\right)!\left(n-s\right)!}f\left(x\right){\left[F\left(x\right)\right]}^{s-1}{\left[1-F\left(x\right)\right]}^{n-s}. \end{array}$$

Using the binomial theorem, we have

fs:n(x)=n!(s-1)!(n-s)!∑w=0n-s(-1)w(n-sw)f(x)[F(x)]w+s-1.

Substituting by Eqs (5) and (6) and using the binomial theorem, we get

fs:n(x)=n!(s-1)!(n-s)!∑w=0n-s∑v=0w+s-1(-1)w+v+1(n-sw)(w+s-1v)αv+1(1-α)w+slog(α)×abcλcxc-1e-(λx)c(1-e-(λx)c)a-1[1-(1-e-(λx)c)a]b-1×α-(v+1)[1-(1-e-(λx)c)a]b.

Then, using the series expansion in Eq (9) and applying the binomial theorem in Eq (10) twice, we obtain

fs:n(x)=n!(s-1)!(n-s)!∑w=0n-s∑v=0w+s-1∑k=0∞∑j=0∞∑i=0∞(n-sw)(w+s-1v)(b(k+1)-1j)(a(j+1)-1i)×(-1)w+v+k+j+i+1k!αv+1(v+1)k(1-α)w+s(log(α))k+1abcλcxc-1e-(i+1)(λx)c. (19)

## 4 Parameter estimation for alpha power Kumaraswamy Weibull distribution

The maximum likelihood method is applied to obtain the estimation for the parameters of APKumW distribution. That is, if we have a random sample *x*_1_, *x*_2_, …, *x*_*n*_ from the APKumW distribution, with the unknown vector of parameter ***θ*** = (*a*, *b*, *c*, λ, *α*), then the log-likelihood function (*ℓ*) can be defined as

$$\begin{array}{cc} \ell = & n\text{log}\;\left(\frac{\alpha \text{log}\left(\alpha \right)}{\alpha -1}\right)+n\;\text{log}\left(abc\lambda^{c}\right)+\left(c-1\right)\sum_{i=1}^{n}\text{log}\left(x_{i}\right)-\sum_{i=1}^{n}{\left(\lambda x_{i}\right)}^{c}+\left(a-1\right)\sum_{i=1}^{n}\text{log}\left(1-e^{-{\left(x_{i}\lambda \right)}^{c}}\right)+\\ & \;\;\left(b-1\right)\sum_{i=1}^{n}\text{log}\left(1-{\left(1-e^{-{\left(x_{i}\lambda \right)}^{c}}\right)}^{a}\right)-\text{log}\left(\alpha \right)\sum_{i=1}^{n}{\left[1-{\left(1-e^{-{\left(\lambda x_{i}\right)}^{c}}\right)}^{a}\right]}^{b}. \end{array}$$ (20)

The associated nonlinear equations for the partial derivative of *ℓ* with respect to each parameter, are given as

$$\begin{array}{cc} \frac{\partial \ell }{\partial a} & =\frac{n}{a}+\sum_{i=1}^{n}\text{log}\left(1-e^{-{\left(\lambda x_{i}\right)}^{c}}\right)\{1-\frac{{\left(1-e^{-{\left(\lambda x\right)}^{c}}\right)}^{a}}{1-{\left(1-e^{-{\left(\lambda x\right)}^{c}}\right)}^{a}}[b\left(1-\text{log}\left(\alpha \right){\left[1-{\left(1-e^{-{\left(\lambda x\right)}^{c}}\right)}^{a}\right]}^{b}\right)-1]\}, \end{array}$$ (21)

$$\begin{array}{c} \frac{\partial \ell }{\partial b}=\frac{n}{b}+\sum_{i=1}^{n}\text{log}[1-{\left(1-e^{-{\left(\lambda x\right)}^{c}}\right)}^{a}][1-\text{log}\left(\alpha \right){\left[1-{\left(1-e^{-{\left(\lambda x\right)}^{c}}\right)}^{a}\right]}^{b}], \end{array}$$ (22)

$$\begin{array}{cc} \frac{\partial \ell }{\partial c} & =\frac{n}{c}\left(1+c\;\text{log}\left(\lambda \right)\right)+\sum_{i=1}^{n}\text{log}\left(x_{i}\right)-\sum_{i=1}^{n}{\left(\lambda x_{i}\right)}^{c}\;\text{log}\left(\lambda x_{i}\right)\{1-\frac{e^{-{\left(\lambda x\right)}^{c}}}{1-e^{-{\left(\lambda x\right)}^{c}}}[a-1+\frac{a{\left(1-e^{-{\left(\lambda x\right)}^{c}}\right)}^{a}}{1-{\left(1-e^{-{\left(\lambda x\right)}^{c}}\right)}^{a}}\times \\ & \;\;\;\;\;\;\;\;\;\;\;\;\left(b\left(1+\text{log}\left(\alpha \right){\left[1-{\left(1-e^{-{\left(\lambda x\right)}^{c}}\right)}^{a}\right]}^{b}\right)-1\right)]\}, \end{array}$$ (23)

$$\begin{array}{cc} \frac{\partial \ell }{\partial \lambda } & =\frac{nc}{\lambda }-c\lambda^{c-1}\sum_{i=1}^{n}x_{i}^{c}\{1-\frac{e^{-{\left(\lambda x\right)}^{c}}}{1-e^{-{\left(\lambda x\right)}^{c}}}[a-1-\frac{a{\left(1-e^{-{\left(\lambda x\right)}^{c}}\right)}^{a}}{1-{\left(1-e^{-{\left(\lambda x\right)}^{c}}\right)}^{a}}\times \\ & \left(b\left(1-\text{log}\left(\alpha \right){\left[1-{\left(1-e^{-{\left(\lambda x\right)}^{c}}\right)}^{a}\right]}^{b}\right)-1\right)]\}, \end{array}$$ (24)

and

$$\begin{array}{c} \frac{\partial \ell }{\partial \alpha }=\frac{1}{\alpha }[\frac{n}{\text{log}\left(\alpha \right)}-\frac{n}{\alpha -1}-\sum_{i=1}^{n}{\left[1-{\left(1-e^{-{\left(\lambda x_{i}\right)}^{c}}\right)}^{a}\right]}^{b}]. \end{array}$$ (25)

Then, the MLEs of the unknown parameters can be obtained by equating the equations from Eqs (21) to (25) to zero and solving them simultaneously. Particularly, a numerical iterative approach, such as the Newton-Raphson algorithm should be applied to solve these equations. Alternatively, any software like R, might be used to maximise Eq (20) directly and obtain the MLEs.

Studying survival times often results in the presence of censored observations, meaning there are incomplete observations of the period of interest. Right censoring technique is applied in medical studies when some patients lost to follow up and their exact occurrence time cannot be determined. The most common form of right censoring, which is encountered in survival analysis, is type I right censoring. A study of this type occurs when it is conducted over a specified period of time that will end before all units have failed. To illustrate, consider a study for a random sample of n patient in which, each patient is assigned a censoring time *Y*_*i*_;*i* = 1, …, *n*, that is the time between entry and the end of the study and where *X*_*i*_;*i* = 1, …, *n*, be the failure time of the *i*^*th*^ patient. These *X*_*i*_’s and *Y*_*i*_’s are supposed to be independent and follow the APKumW distribution in Eq (6) and a non-informative distribution, respectively. For *T*_*i*_ = min(*X*_*i*_, *Y*_*i*_), the pair (*T*_*i*_, *δ*_*i*_) is observed such that

$$\delta_{i}=\{\begin{array}{c} 1\;\text{if}\;\text{failure}\;\text{has}\;\text{occurred}\\ 0\;\text{if}\;\text{censoring}\;\text{has}\;\text{occurred} \end{array}$$

Then, the log-likelihood function (*ℓ*) will be

$$\begin{array}{c} \ell =\sum_{i=1}^{n}\delta_{i}\;\text{log}\left[f\left(t_{i}\right)\right]+\sum_{i=1}^{n}\left(1-\delta_{i}\right)\text{log}\left[SF\left(t_{i}\right)\right], \end{array}$$ (26)

where *f*(.) and *SF*(.) are respectively defined in Eqs (6) and (7). In order to obtain the MLEs, the log-likelihood in Eq (26) can be maximized numerically.

## 5 Simulation study

Some simulation studies are conducted to evaluate the performance of the MLEs for the five parameters of APKumW distribution. The simulation is considered over a number of iterations equal to *nsim* = 1000, for different sample sizes *n* with the following cases for the true parameters *θ*_*tr*_

- **Case I**: *a* = 0.5, *b* = 0.6, *c* = 1.3, λ = 0.2, *α* = 0.1
- **Case II**: *a* = 0.8, *b* = 2.7, *c* = 3.6, λ = 2.4, *α* = 1.2
- **Case III**: *a* = 1.3, *b* = 0.4, *c* = 1.6, λ = 0.5, *α* = 0.2
- **Case IV**: *a* = 2.2, *b* = 1.6, *c* = 1.4, λ = 0.07, *α* = 0.03

The MLEs for each estimator $\hat{\theta }$ can be evaluated using an accuracy measurement, such as the root mean squared error (RMSE) that can be calculated as follows

$$\begin{array}{c} RMSE\left(\hat{\theta }\right)=\sqrt{\frac{\sum_{i=1}^{nsim}{\left({\hat{\theta }}_{i}-\theta_{tr}\right)}^{2}}{nsim}} \end{array}$$ (27)

All estimation results are obtained using the “optim” function in R software. Table 2 shows the results for the MLEs of the parameters of APKumW along with their corresponding RMSE. Generally, it can be seen from this table, that the MLEs are more closer to the true values of the parameters as the sample size increased. In addition, RMSE became smaller as sample size *n* increased, indicating that the estimates are consistent. These results demonstrate that maximum likelihood method is effective at estimating the parameters of the proposed distribution.

**Table 2:** Simulation study: APKumW parameter estimates, together with the RMSE for three different cases with different sample sizes.

		Case I		Case II		Case III		Case IV	
		MLE	RMSE	MLE	RMSE	MLE	RMSE	MLE	RMSE
*n* = 25	*a*	0.7374	1.1311	1.1366	1.7467	3.1581	12.3097	4.1684	5.8308
	*b*	1.3078	1.7491	2.9682	4.1624	0.4752	0.6268	1.5157	1.8984
	*c*	2.2590	1.8346	6.1786	5.0080	2.0867	1.7713	2.2103	2.1365
	λ	0.4063	0.8406	2.9813	1.7717	1.0853	1.3514	0.1890	0.2221
	*α*	0.4441	1.7620	2.8268	8.8754	0.8573	1.8160	0.5149	1.0372
*n* = 50	*a*	0.5936	0.7352	0.8191	0.8192	2.2807	4.3226	3.7718	4.3232
	*b*	1.1829	1.3862	3.0076	3.9600	0.4590	0.4819	1.4895	1.8195
	*c*	1.8486	1.2120	5.6346	3.9938	1.7671	0.9930	1.7707	1.1602
	λ	0.3280	0.6997	2.7180	1.0426	0.8880	0.9771	0.1720	0.1899
	*α*	0.3669	1.0412	2.9617	6.8317	0.6719	1.3418	0.4554	0.9540
*n* = 100	*a*	0.5002	0.3808	0.7360	0.5757	1.7846	1.3917	3.4064	3.2416
	*b*	1.0501	0.9617	2.8792	2.8078	0.4423	0.4725	1.5827	1.5955
	*c*	1.7149	0.9487	5.2997	3.2607	1.5933	0.4265	1.5137	0.6138
	λ	0.2617	0.4597	2.5268	0.6980	0.7591	0.6132	0.1450	0.1497
	*α*	0.2640	0.6310	2.6456	5.7300	0.6315	1.2849	0.3413	0.7541
*n* = 500	*a*	0.4883	0.1427	0.7330	0.2806	1.5301	0.6641	2.6718	1.3610
	*b*	0.8331	0.5439	3.0105	1.8025	0.4243	0.3008	1.6417	0.9258
	*c*	1.4369	0.4402	4.4465	1.9672	1.5428	0.2977	1.3714	0.3012
	λ	0.2056	0.1635	2.4009	0.4645	0.6278	0.3305	0.0939	0.0577
	*α*	0.1791	0.2369	1.9772	2.8766	0.5035	0.8366	0.1442	0.3296
*n* = 1000	*a*	0.4891	0.1038	0.7486	0.2159	1.4622	0.4818	2.4720	0.8942
	*b*	0.7738	0.4584	2.8873	1.3060	0.4324	0.2165	1.6791	0.7932
	*c*	1.3883	0.3229	4.1520	1.4735	1.5478	0.2613	1.3679	0.2323
	λ	0.1986	0.1182	2.3915	0.3327	0.5743	0.2204	0.0809	0.0341
	*α*	0.1589	0.1963	1.5851	1.6256	0.4032	0.5784	0.0803	0.1853

## 6 Applications

Six real datasets for cancer patients are fitted using the APKumW distribution. The results obtained using the APKumW distribution are compared against the corresponding ones achieved with the application of the following

The Weibull distribution with the following pdf

$$f\left(x\right)=\frac{c}{\lambda^{c}}x^{c-1}e^{-{\left(\frac{x}{\lambda }\right)}^{c}}.$$

The Exponenetiated generalized Weibull (EGW) distribution [46] with the following pdf

$$f\left(x\right)=ab\frac{c}{\lambda^{c}}x^{c-1}e^{-a{\left(\frac{x}{\lambda }\right)}^{c}}{\left[1-e^{-a{\left(\frac{x}{\lambda }\right)}^{c}}\right]}^{b-1}.$$

The Beta Weibull (BW) distribution [4] with the following pdf

$$f\left(x\right)=\frac{\Gamma \left(a+b\right)}{\Gamma \left(a\right)\Gamma \left(b\right)}\frac{c}{\lambda^{c}}x^{c-1}e^{-b{\left(\frac{x}{\lambda }\right)}^{c}}{\left[1-e^{-{\left(\frac{x}{\lambda }\right)}^{c}}\right]}^{a-1}.$$

The KumW distribution [21] with the following pdf

$$f\left(x\right)=ab\frac{c}{\lambda^{c}}x^{c-1}e^{-{\left(\frac{x}{\lambda }\right)}^{c}}{\left(1-e^{-{\left(\frac{x}{\lambda }\right)}^{c}}\right)}^{a-1}{\left[1-{\left(1-e^{-{\left(\frac{x}{\lambda }\right)}^{c}}\right)}^{a}\right]}^{b-1}.$$

The exponentiated Kumaraswamy Weibull (EKumW) distribution [47] with the following pdf

$$\begin{array}{c} f\left(x\right)=\alpha abc\lambda^{c}x^{c-1}e^{-{\left(\lambda x\right)}^{c}}{\left(1-e^{-{\left(\lambda x\right)}^{c}}\right)}^{a-1}{\left[1-{\left(1-e^{-{\left(\lambda x\right)}^{c}}\right)}^{a}\right]}^{b-1}\times \\ {\left\{1-{\left[1-{\left(1-e^{-{\left(\lambda x\right)}^{c}}\right)}^{a}\right]}^{b}\right\}}^{\alpha -1}. \end{array}$$

The alpha power Weibull (APW) distribution [32] with the following pdf

$$f\left(x;\theta \right)=\{\begin{array}{cc} \begin{array}{ccc} \frac{log\left(\alpha \right)}{\alpha -1} & c\lambda x^{c-1} & e^{-\lambda x^{c}}\alpha^{1-e^{-\lambda x^{c}}} \end{array}\;;\; & \text{if}\;\alpha >0,\alpha \neq 1\\ c\lambda x^{c-1}e^{-\lambda x^{c}}\;\;\;\;\;;\; & \text{if}\;\alpha =1. \end{array}$$

**Fig 2:**
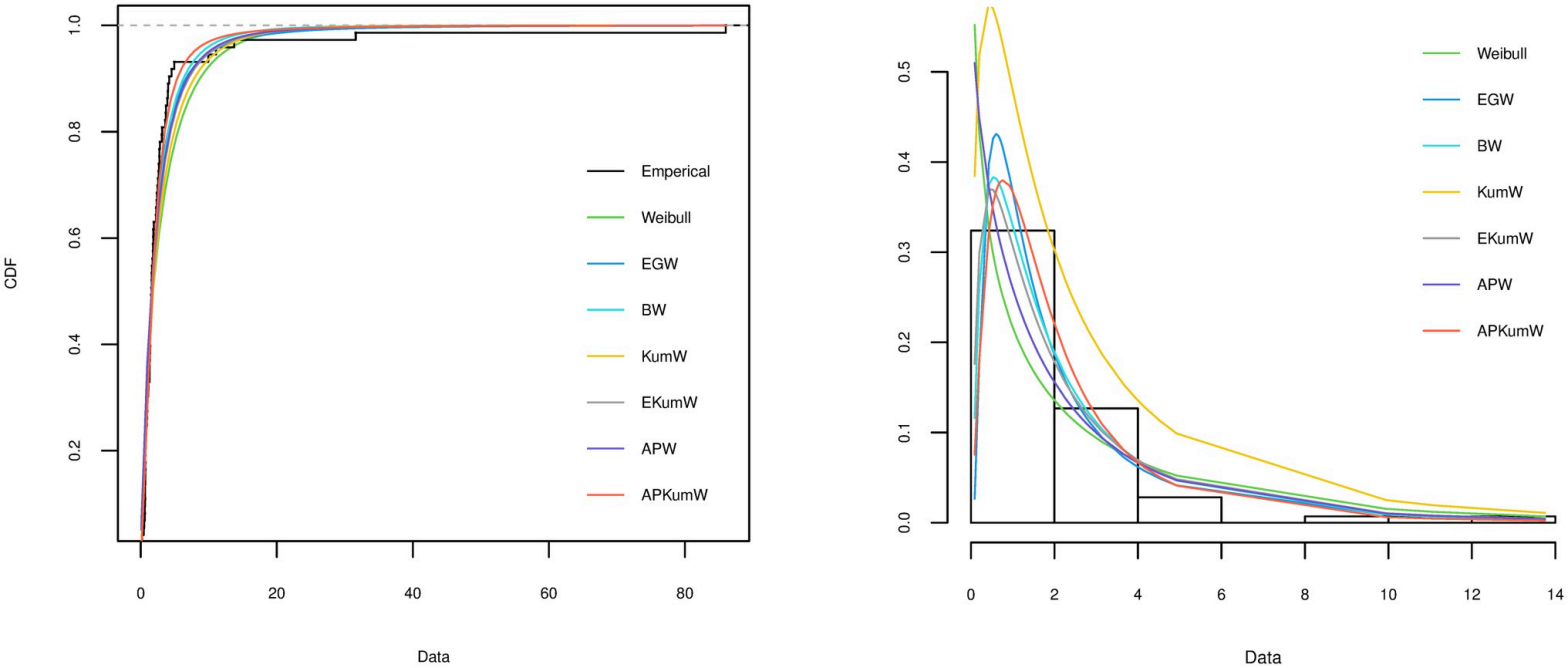
Theoretical and empirical cdf and pdf comparison of the acute bone cancer data.

**Fig 3:**
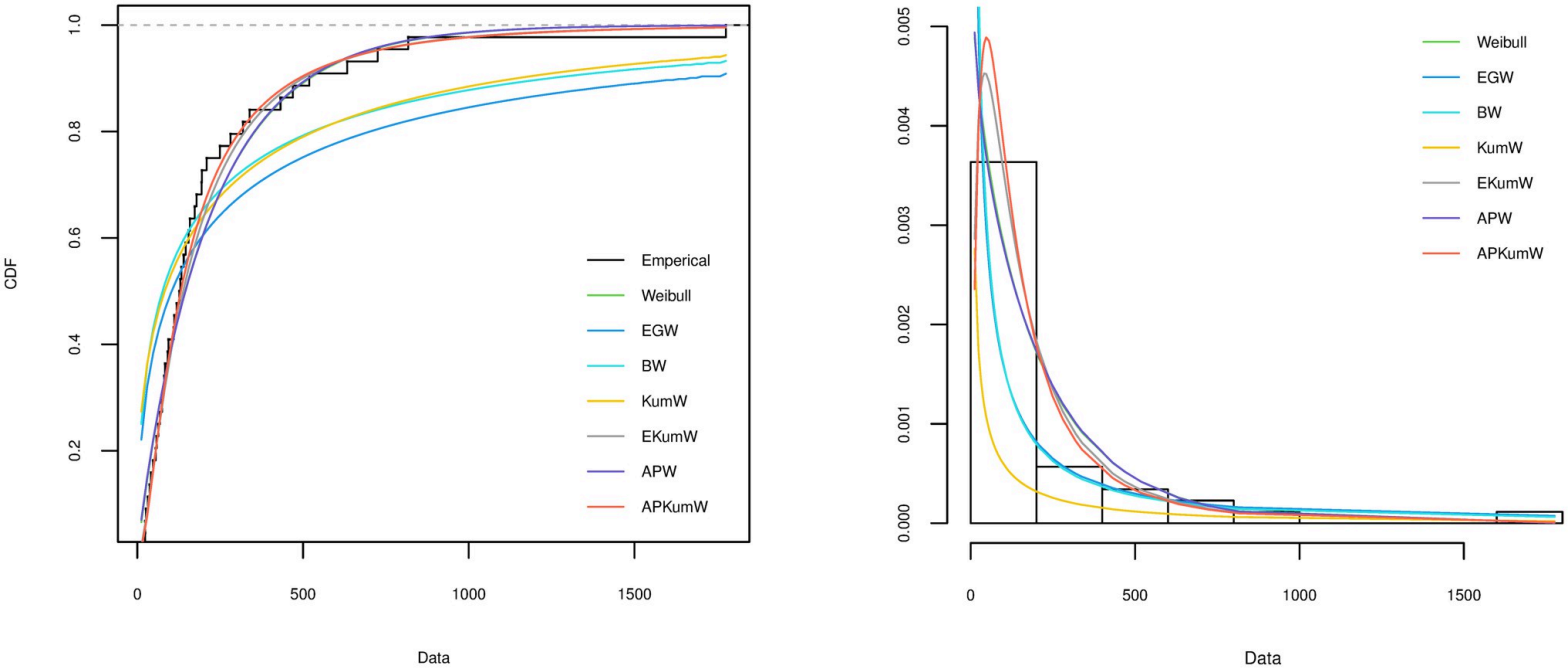
Theoretical and empirical cdf and pdf comparison of the head and Neck cancer data.

**Fig 4:**
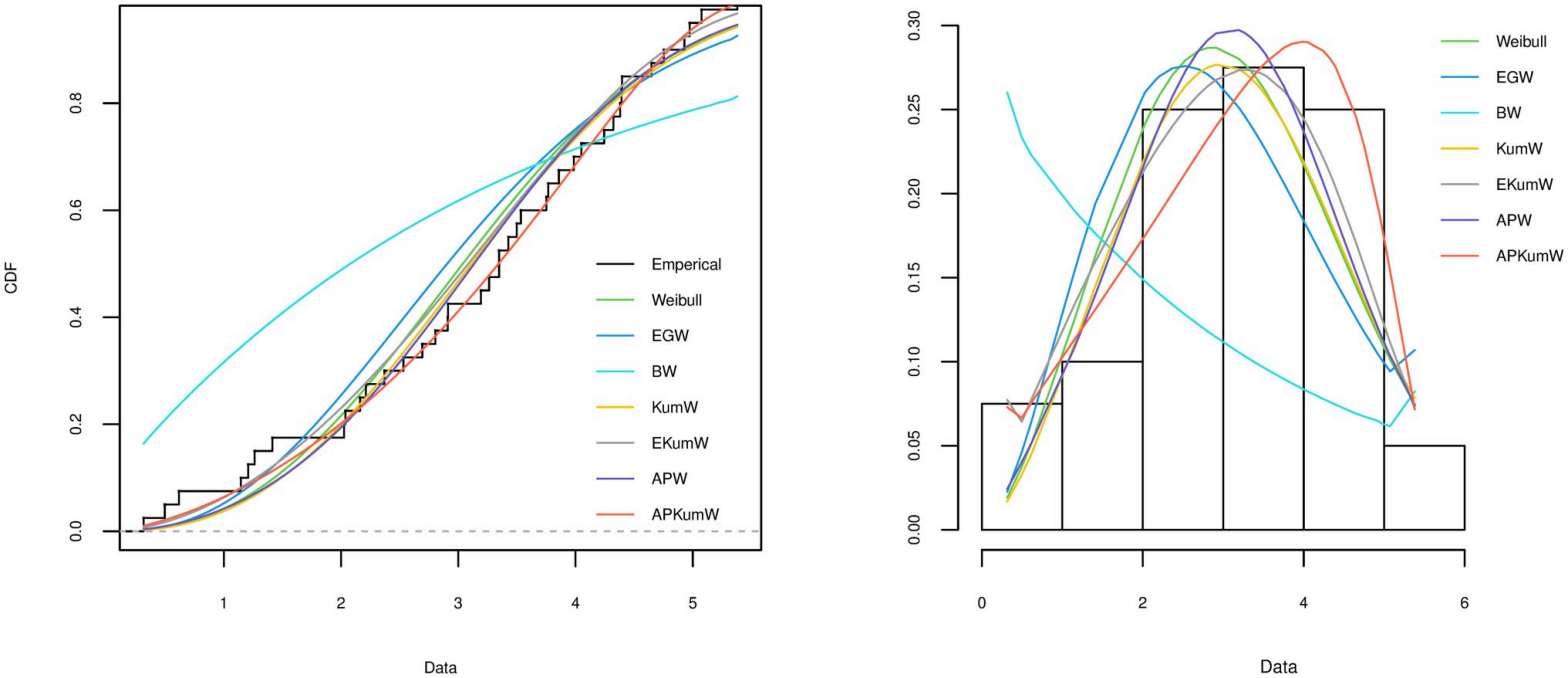
Theoretical and empirical cdf and pdf comparison of the blood cancer data.

**Fig 5:**
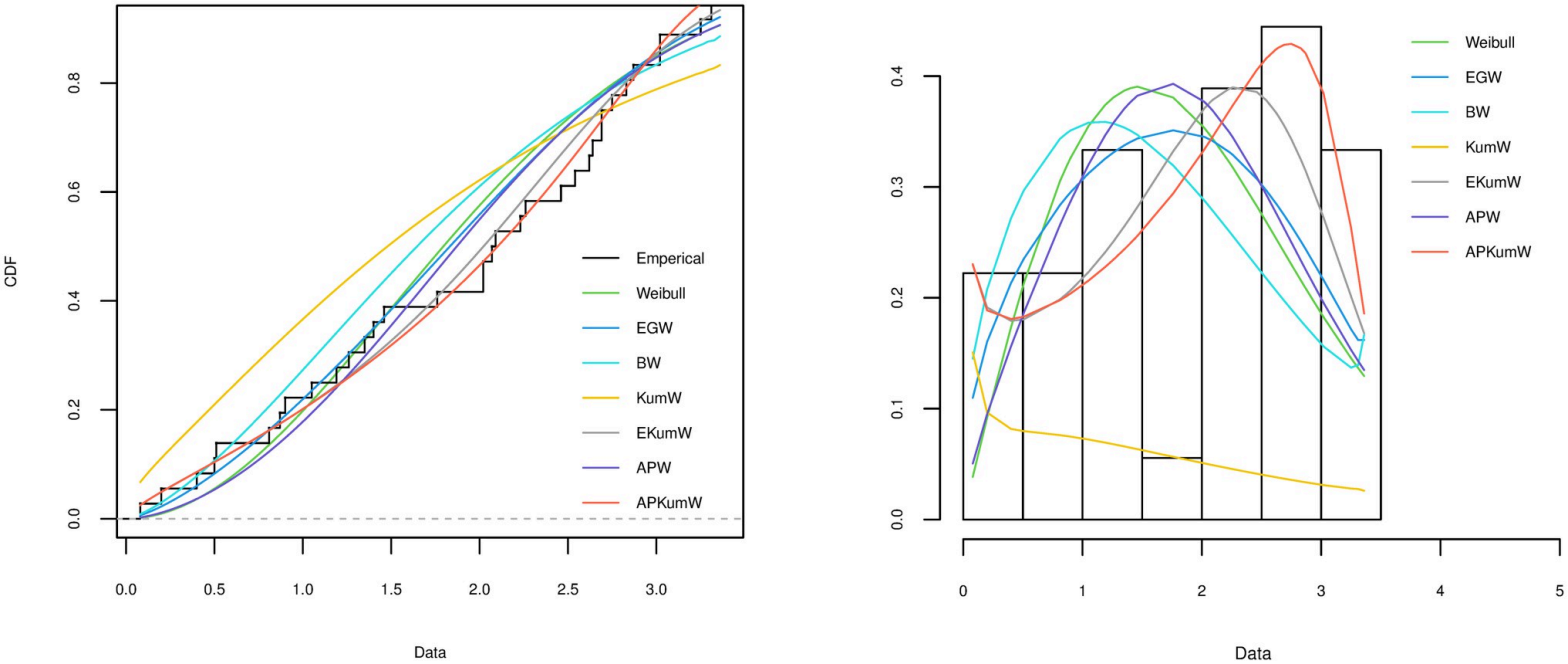
Theoretical and empirical cdf and pdf comparison of the bladder cancer I data.

**Fig 6:**
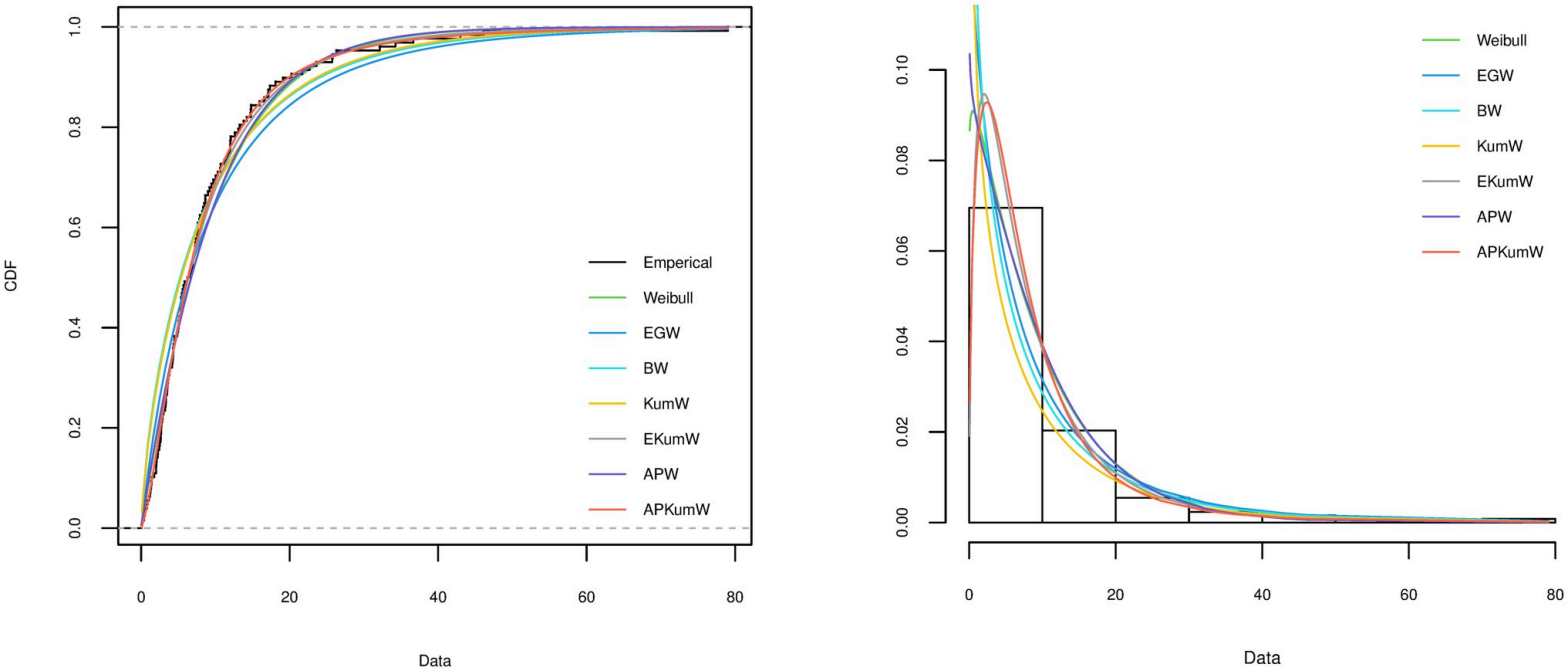
Theoretical and empirical cdf and pdf comparison of the bladder cancer II data.

A variety of tools can be applied for comparing different competing distribution for a specific dataset and choosing the best model for the fitting. To investigate the goodness-of-fit for the compared distribution, Akaike Information Criterion (AIC) and Kolmogorov–Smirnov (KS) along with its P value are considered in order to choose the best distribution. The better distribution is which corresponds to the lower values of AIC, KS and highest P value of KS statistic. The plots of the estimated cdf for each of the distributions are compared with the plot of the empirical cdf. Also, the histogram of the observed frequencies is compared with the plots of the expected frequencies for each fitted distribution. The MLEs of the parameters for all the five datasets along with their SEs (in parentheses) and the corresponding goodness-of-fit criteria for all the competing models are respectively presented in Tables 3–7. Additionally, Figs 2–6 display on lefts the empirical and the fitted cdfs, and on rights the fitted pdfs with histogram of the observed datasets in Tables 3–7.

**Table 3:** MLEs, (SEs) for the parameters and associated goodness of fit statistics for the acute bone cancer data.

Distribution	MLE and SE	AIC	KS	P value
APKumW	*α* = 0.0046 (0.0055), *a* = 5.0887 (2.1344), *b* = 0.4137 (0.3181), *c* = 0.5358 (0.1424), λ = 1.3007 (0.7691)	291.7005	0.0680	0.8888
Weibull	*c* = 0.7656 (0.0568), λ = 2.9260 (0.4761)	326.8033	0.1887	0.0111
EGW	*a* = 2.7262 (4.3589), *b* = 80.5514 (119.2631), *c* = 0.2353 (0.0755), λ = 0.15070 (0.9666)	294.0796	0.0924	0.5612
BW	*a* = 59.2646 (35.3442), *b* = 62.3944 (32.4899), *c* = 0.1262 (0.0363), λ = 41.4347 (42.1657)	298.9643	0.0988	0.4747
KumW	*a* = 2.7498 (0.0095), *b* = 0.3506 (0.0423), *c* = 0.6483 (0.0035), λ = 0.3447 (0.0052)	311.4273	0.1470	0.0853
EKumW	*a* = 1.273 (0.4415), *b* = 1.9974 (1.0322), *c* = 0.4002 (0.0658), λ = 1.0345 (1.066), *α* = 5.4855 (2.0861)	302.2774	0.1168	0.272
APW	*c* = 0.9218 (0.078), λ = 0.0791 (0.0141), *α* = 0.0021 (0.001)	309.0348	0.1884	0.0112

**Table 4:** MLEs, (SEs) for the parameters and associated goodness of fit statistics for the head and Neck cancer data.

Distribution	MLE and SE	AIC	KS	P value
APKumW	*a* = 4.8428 (3.2155), *b* = 0.6523 (0.7809), *c* = 0.5719 (0.2531), λ = 0.026 (0.0106), *α* = 0.3173 (1.0202)	565.1112	0.0751	0.9492
Weibull	*c* = 0.9386 (0.1007), λ = 213.6881 (36.2325)	567.6877	0.1267	0.4435
EGW	*a* = 0.0784 (0.0131), *b* = 1.6582 (0.3456), *c* = 0.3437 (0.0022), λ = 0.0513 (0.0028)	602.3591	0.2687	0.0027
BW	*a* = 2.3728 (1.1206), *b* = 0.0759 (0.0119), *c* = 0.3999 (0.002), λ = 0.2225 (0.0025)	602.5257	0.3075	0.0003
KumW	*a* = 0.3532 (0.1743), *b* = 0.0782 (0.0122), *c* = 0.4911 (0.0025), λ = 1.2577 (0.0025)	604.0207	0.3142	0.0002
EKumW	*a* = 7.8983 (2.4909), *b* = 6.8318 (3.9697), *c* = 0.2203 (0.0377), λ = 0.0541 (0.0336), *α* = 1.8148 (1.1851)	566.0263	0.0973	0.7625
APW	*c* = 0.8779 (0.077), λ = 0.0105 (0.0089), *α* = 1.6918 (2.2826)	570.2769	0.1277	0.4342

**Table 5:** MLEs, (SEs) for the parameters and associated goodness of fit statistics for the blood cancer data.

Distribution	MLE and SE	AIC	KS	P value
APKumW	*a* = 0.1192 (0.0576), *b* = 1.493 (0.2111), *c* = 12.8763 (0.0026), λ = 0.1888 (0.0025), *α* = 3.2018 (8.3932)	139.4392	0.0625	0.9976
Weibull	*c* = 2.4993 (0.3370), λ = 3.5183 (0.2316)	143.1159	0.1185	0.6284
EGW	*a* = 0.0814 (0.0147), *b* = 1.4848 (0.3171), *c* = 1.7819 (0.0025), λ = 0.7165 (0.0025)	149.7559	0.1495	0.3330
BW	*a* = 0.3617 (0.1295), *b* = 0.0431 (0.0071), *c* = 1.008 (0.0023), λ = 0.1524 (0.0023)	185.7482	0.3171	0.0006
KumW	*a* = 0.9562 (0.0734), *b* = 0.0838 (0.0133), *c* = 2.5959 (0.0083), λ = 1.3823 (0.0083)	146.7387	0.0975	0.8411
EKumW	*a* = 3.2765 (0.0021), *b* = 1.3117 (0.0677), *c* = 4.1917 (0.0021), λ = 0.2253 (0.002), *α* = 0.1367 (0.0218)	142.2623	0.1039	0.7806
APW	*c* = 2.101 (0.4359), λ = 0.1058 (0.0827), *α* = 5.3518 (6.7462)	143.3641	0.0919	0.888

**Table 6:** MLEs, (SEs) for the parameters and associated goodness of fit statistics for the bladder cancer I data.

Distribution	MLE and SE	AIC	KS	P value
APKumW	*a* = 0.0692 (0.0859), *b* = 0.8434 (0.1146), *c* = 9.3634 (0.0079), λ = 0.3289 (0.0075), *α* = 8.4765 (35.1643),	100.1924	0.0806	0.9736
Weibull	*c* = 1.9570 (0.2816), λ = 2.1645 (0.1918)	106.7741	0.165	0.2753
EGW	*a* = 0.0478 (0.0141), *b* = 0.3927 (0.0748), *c* = 3.5985 (0.0036), λ = 1.2523 (0.0043)	104.5355	0.1490	0.4015
BW	*a* = 0.8648 (0.3929), *b* = 0.0587 (0.0101), *c* = 1.6055 (0.0016), λ = 0.3582 (0.0016)	110.6557	0.1995	0.1139
KumW	*a* = 0.2273 (0.0026), *b* = 0.0487 (0.0081), *c* = 1.2286 (0.0014), λ = 0.1863 (0.0013)	121.6362	0.2092	0.0855
EKumW	*a* = 0.2692 (0.1951), *b* = 0.1116 (0.0855), *c* = 3.7307 (0.9426), λ = 0.6668 (0.1644), *α* = 0.7241 (0.3537)	104.0068	0.0904	0.9302
APW	*c* = 1.6796 (0.3636), λ = 0.3915 (0.2099), *α* = 4.5688 (5.7946)	107.2964	0.1406	0.4751

**Table 7:** MLEs, (SEs) for the parameters and associated goodness of fit statistics for the bladder cancer II data.

Distribution	MLE and SE	AIC	KS	P value
APKumW	*a* = 1.4958 (0.8164), *b* = 0.3964 (1.1823), *c* = 1.0028 (0.4566), λ = 0.1139 (0.2304), *α* = 0.0168 (0.0409),	829.509	0.0370	0.9947
Weibull	*c* = 1.0478 (0.0676), λ = 9.5607 (0.8529)	832.1738	0.0700	0.5570
EGW	*a* = 0.1608 (0.0169), *b* = 1.4844 (0.185), *c* = 0.7023 (0.0026), λ = 0.4745 (0.0026)	841.2811	0.1091	0.0947
BW	*a* = 0.8742 (0.1428), *b* = 0.163 (0.0157), *c* = 0.8088 (0.0026), λ = 0.9306 (0.0026)	854.7262	0.1755	0.0007
KumW	*a* = 0.8027 (0.1796), *b* = 0.1707 (0.0164), *c* = 0.8367 (0.0026), λ = 1.079 (0.0026)	852.5814	0.1664	0.0017
EKumW	*a* = 1.2965 (0.6309), *b* = 0.7662 (0.7314), *c* = 0.6749 (0.1550), λ = 0.4087 (0.4881), *α* = 2.1286 (1.1644)	831.4566	0.0418	0.9786
APW	*c* = 0.9553 (0.088), λ = 0.1449 (0.0656), *α* = 2.1243 (1.8644)	834.9494	0.0725	0.5115

### 6.1 Acute bone cancer dataset

[48] considered a simulated data represents the survival times (in days) of 73 patients who diagnosed with acute bone cancer, as follows: 0.09, 0.76, 1.81, 1.10, 3.72, 0.72, 2.49, 1.00, 0.53, 0.66, 31.61, 0.60, 0.20, 1.61, 1.88, 0.70, 1.36, 0.43, 3.16, 1.57, 4.93, 11.07, 1.63, 1.39, 4.54, 3.12, 86.01, 1.92, 0.92, 4.04, 1.16, 2.26, 0.20, 0.94, 1.82, 3.99, 1.46, 2.75, 1.38, 2.76, 1.86, 2.68, 1.76, 0.67, 1.29, 1.56, 2.83, 0.71, 1.48, 2.41, 0.66, 0.65, 2.36, 1.29, 13.75, 0.67, 3.70, 0.76, 3.63, 0.68, 2.65, 0.95, 2.30, 2.57, 0.61, 3.93, 1.56, 1.29, 9.94, 1.67, 1.42, 4.18, 1.37. This medical dataset is considered and analyzed using the APKumW and the competitive models.

### 6.2 Head and Neck cancer dataset

Survival time for 44 patients diagnosed by Head and Neck cancer disease from [49] and analyzed recently by [50] is considered. The dataset are: 12.20, 23.56, 23.74, 25.87, 31.98, 37, 41.35, 47.38, 55.46, 58.36, 63.47, 68.46, 78.26, 74.47, 81.43, 84, 92, 94, 110, 112, 119, 127, 130, 133, 140, 146, 155, 159, 173, 179, 194,195, 209, 249, 281, 319, 339, 432, 469, 519, 633, 725, 817, 1776.

### 6.3 Blood cancer dataset

This data consists of the life time (in years) of a 40 blood cancer (leukemia) patients from one of Ministry of health hospitals in Saudi Arabia reported in [51]. This actual data are: 0.315, 0.496, 0.616, 1.145, 1.208, 1.263, 1.414, 2.025, 2.036, 2.162, 2.211, 2.370, 2.532, 2.693, 2.805, 2.910, 2.912, 3.192, 3.263, 3.348, 3.348, 3.427, 3.499, 3.534, 3.767, 3.751, 3.858, 3.986, 4.049, 4.244, 4.323, 4.381, 4.392, 4.397, 4.647, 4.753, 4.929, 4.973, 5.074, 5.381.

### 6.4 Bladder cancer I dataset

The dataset on the remission times (in months) of a 36 bladder cancer patients reported in [52] and given by: 0.08, 0.2, 0.4, 0.5,0.51, 0.81, 0.87, 0.9, 1.05, 1.19, 1.26, 1.35, 1.4, 1.46, 1.76, 2.02, 2.02, 2.07, 2.09, 2.23, 2.26, 2.46, 2.54, 2.62, 2.64, 2.69, 2.69, 2.75, 2.83, 2.87, 3.02, 3.02, 3.25, 3.31, 3.36, 3.36.

### 6.5 Bladder cancer II dataset

This application is from [53] and it is about the remission times (in months) of a 128 patients suffering from bladder cancer. This data has been analyzed recently in many papers, such as [54, 55]. The dataset values are as follows: 0.08, 2.09, 3.48, 4.87, 6.94, 8.66, 13.11, 23.63, 0.20, 2.23, 3.52, 4.98, 6.97, 9.02, 13.29, 0.40, 2.26, 3.57, 5.06, 7.09, 9.22, 13.80, 25.74, 0.50, 2.46, 3.64, 5.09, 7.26, 9.47, 14.24, 25.82, 0.51, 2.54, 3.70, 5.17, 7.28, 9.74, 14.76, 26.31, 0.81, 2.62, 3.82, 5.32, 7.32, 10.06, 14.77, 32.15, 2.64, 3.88, 5.32, 7.39, 10.34, 14.83, 34.26, 0.90, 2.69, 4.18, 5.34, 7.59, 10.66, 15.96, 36.66, 1.05, 2.69, 4.23, 5.41, 7.62, 10.75, 16.62, 43.01, 1.19, 2.75, 4.26, 5.41, 7.63, 17.12, 46.12, 1.26, 2.83, 4.33, 5.49, 7.66, 11.25, 17.14, 79.05, 1.35, 2.87, 5.62, 7.87, 11.64, 17.36, 1.40, 3.02, 4.34, 5.71, 7.93, 11.79, 18.10, 1.46, 4.40, 5.85, 8.26, 11.98, 19.13, 1.76, 3.25, 4.50, 6.25, 8.37, 12.02, 2.02, 3.31, 4.51, 6.54, 8.53, 12.03, 20.28, 2.02, 3.36, 6.76, 12.07, 21.73, 2.07, 3.36, 6.93, 8.65, 12.63, 22.69.

On the basis of the results presented in Tables 3–7, it can be seen that APKumW is the best model of all fitted distributions, resulting in the lowest values for AIC, KS and highest P values of KS statistic across all datasets. Besides, Figs 2–6 demonstrate the adequacy of the suggested APKumW distribution due to its closed fit to the observed cancer datasets. Thus, it can be concluded that the APKumW would be preferred to the other distributions applied to the considered cancer datasets.

### 6.6 Censored dataset

Listed below are the ordered remission times (in months) of a random sample of 137 bladder cancer patients from [53]: 0.08, 2.09, 3.48, 4.87, 6.94, 8.66, 13.11, 23.63, 0.20, 2.23, 3.52, 4.98, 6.97, 9.02, 13.29, 24.80*, 0.40, 2.26, 3.57, 5.06, 7.09, 9.22, 13.80, 25.74, 0.50, 2.46, 3.64, 5.09, 7.26, 9.47, 14.24, 25.82, 0.51, 2.54, 3.70, 5.17, 7.28, 9.74, 14.76, 26.31, 0.81, 2.62, 3.82, 5.32, 7.32, 10.06, 14.77, 32.15, 0.87*, 2.64, 3.88, 5.32, 7.39, 10.34, 14.83, 34.26, 0.90, 2.69, 4.18, 5.34, 7.59, 10.66, 15.96, 36.66, 1.05, 2.69, 4.23, 5.41, 7.62, 10.75, 16.62, 43.01, 1.19, 2.75, 4.26, 5.41, 7.63, 10.86*, 17.12, 46.12, 1.26, 2.83, 4.33, 5.49, 7.66, 11.25, 17.14, 79.05, 1.35, 2.87, 4.33*, 5.62, 7.87, 11.64, 17.36, 1.40, 3.02, 4.34, 5.71, 7.93, 11.79, 18.10, 1.46, 3.02*, 4.40, 5.85, 8.26, 11.98, 19.13, 1.76, 3.25, 4.50, 6.25, 8.37, 12.02, 19.36*, 2.02, 3.31, 4.51, 6.54, 8.53, 12.03, 20.28, 2.02, 3.36, 4.65*, 6.76, 8.60*, 12.07, 21.73, 2.07, 3.36, 4.70*, 6.93, 8.65, 12.63, 22.69. The asterisk * means censored data.

Table 8 shows the MLEs, SEs of the unknown parameters of the APKumW distribution for the censored data obtained by maximizing the log-likelihood function in Eq (26). The table also displays the MLEs, SEs of the unknown parameters of the Weibull and exponentiated Kumaraswamy Weibull (EKumW) distributions based on the censored cancer data. As shown by the lowest AIC for the APKumW, it appears that the distribution can fit censored data well.

**Table 8:** MLE, (SE) for the parameters and associated goodness of fit statistics for the censored data.

Distribution	MLE and SE	AIC
APKumW	*a* = 1.4053 (0.6525), *b* = 0.3253 (1.0591), *c* = 1.0518 (0.3953), λ = 0.1309 (0.2918), *α* = 0.0273 (0.0637),	846.8787
Weibull	*c* = 1.0164 (0.0655), λ = 0.1106 (0.0099)	847.7042
EKumW	*a* = 0.4527 (0.1008), *b* = 0.2853 (0.0353), *c* = 1.3493 (0.0028), λ = 0.1782 (0.0023), *α* = 0.9005 (0.1333)	875.5156

## 7 Conclusion

Choosing a suitable model for fitting survival data has been a major concern among researchers. One of the most popular distributions for life-time data is the Weibull distribution. In this paper, the Weibull distribution is extended to provide a new distribution called the APKumW to model life time data. It has different special cases which have been presented in the paper. A number of statistical characteristics of the proposed distribution have been studied, including survival and hazard functions, quantiles, moments, Rényi entropy and order statistics. Inference of parameters for an APKumW was obtained using the method of maximum likelihood. The estimates have been evaluated via different simulation studies. A good performance is observed when the parameters have been estimated using the maximum likelihood method. The applications of statistical distributions are essential for medical research and can have a crucial impact on public health, especially for cancer patients. Thus, the usefulness of this distribution is illustrated through its applications to some real datasets that describe the survival of some cancer patients, including both complete and censored cases. The results indicate the superior performance of the APKumW distribution compared to other competitive distributions by means of different goodness-of-fit criteria. Overall, it is hoped that the proposed APKumW distribution will provide an alternative to other existing distributions available for modeling positive skewed real data in survival analysis, especially for cancer research.

## Abbreviations

AIC: Akaike Information Criterion
APKumW: alpha power Kumaraswamy Weibull
APT: alpha power transformation
APW: alpha power Weibull
BW: Beta Weibull
cdf: cumulative distribution function
EGW: Exponenetiated generalized Weibull
EKumW: exponentiated Kumaraswamy Weibull
KS: Kolmogorov–Smirnov
Kum-G: Kumaraswamy generalized
KumW: Kumaraswamy Weibull
KumW: Kumaraswamy Weibull
MLE: maximum likelihood estimator
pdf: probability density function
RMSE: root mean squared error
SE: standard error
